# Working Conditions and Urinalysis Dipstick Testing among Female Rice Farmers: A Preliminary Cross-Sectional Study

**DOI:** 10.3390/ijerph18178942

**Published:** 2021-08-25

**Authors:** Sara Arphorn, Aniruth Manothum, Kotchakorn Santiwung, Kanograt Pangunta, Kunio Hara, Tomohiro Ishimaru

**Affiliations:** 1 Department of Occupational Health and Safety, Faculty of Public Health, Mahidol University, Bangkok 10400, Thailand; sara.arp@mahidol.ac.th; 2 Department of Industrial Arts, Faculty of Industrial Technology, Lampang Rajabhat University, Lampang 52100, Thailand; 3 Department of Agricultural Extension, Muang, Nan 55000, Thailand; chakorn129@gmail.com; 4 Srinachuen Health Promoting Hospital, Wiangsa, Nan 55110, Thailand; Kanograt05@gmail.com; 5 Department of Occupational Safety and Health Management, School of Health Sciences, University of Occupational and Environmental Health, Japan, Kitakyushu 8078555, Japan; kunio_hara@health.uoeh-u.ac.jp; 6 Department of Environmental Epidemiology, Institute of Industrial Ecological Sciences, University of Occupational and Environmental Health, Kitakyushu 8078555, Japan; ishimaru@med.uoeh-u.ac.jp

**Keywords:** female workers, renal function, rice farmers, urinalysis, working conditions

## Abstract

This cross-sectional study aimed to assess working conditions and renal function among female rice farmers in Nan Province, Thailand. Purposive random sampling was used to select participants who met the inclusion criteria. Data were collected from 65 female rice farmers using in-depth interviews. A walk-through survey was performed to examine hazards in the rice farm and a dipstick test was used for urinalysis. The results demonstrated that all rice farming stages contained risks from physical, chemical, biological and ergonomic hazards and that no measures had been implemented to protect participants’ health from these risks. The screening test results showed low urinary pH (5–6) in 54 women (83.0%), but high urinary specific gravity (1.030) in 25 women (38.5%). Participants’ urine contained leukocytes in 15 women (23.1%), protein in 14 women (21.5%) and blood in 13 women (20%). This study results suggest that farming activities affect female rice farmers’ health and safety. Urinalysis dipstick tests, which can be administered at the local public health promoting hospital, should be used to assess the occupational health status. The results will be useful for health surveillance and follow-up of the participants. These preliminary findings need to be confirmed in a larger study including non-farmers.

## 1. Introduction

More attention is being paid to female workers worldwide. According to the International Labour Organization (ILO), global reports of the gender employment gap show that the current global labor force participation rate for women is up to 49%. The labor force participation rate for female workers in Thailand is 60.5% [1]. This is consistent with statistics for informal workers in Thailand, which shows that there were 9.2 million informal female workers in 2020, an increase from 9.0 million in 2019 [2]. The United Nations has established the 17 Sustainable Development Goals, which call for urgent global action. One key policy is gender equality, which aims to create gender equality—including equality between men and women—in the labor market [3]. In Thailand, the role of women is continually developing. Both public and private organizations are attempting to reduce gender inequality by increasing the role of Thai women in society, for example, by encouraging female leaders in organizational management. Consequently, there are opportunities for Thai women to effectively develop their potential. As a result, Thailand’s ratio of female to male managers is the third highest in Southeast Asia and ranks 64th in the world [4,5]. However, there are little data on the health of female workers, particularly in agriculture. Women in agricultural work are exposed to the same injuries and occupational hazards as men. According to reports on the gender employment gap, women tend to work in low-quality jobs in hazardous conditions and there is little chance that this situation will improve in the near future [1]. In addition, women have substantially greater vulnerability to some diseases than men [6,7]. These factors pose challenges to the health care system and health education of female workers in the work environment is required.

In Thailand, rice is an economically and agriculturally productive crop that provides work and a regular high income. In 2019, Thailand was one of five global rice exporters, exporting 0.378 million tons of rice [8]. Female farmers earn an income for their families and community and are engaged in both rice farming and home care. However, rice farming is a risky occupation characterized by a range of health threats associated with work activities and the working environment; farmers are exposed to physical, chemical, biological and ergonomic health hazards [9] and hazardous energy sources. Such risks are more complex today than in the past because of technologies used to increase agricultural production, such as smart farming and electrical control systems. In addition, to increase agricultural production and in response to high competition in a difficult farming environment, many pesticides are used, which have long-term health risks for rice farmers [10].

In Thailand, public health promoting hospitals (HPHs) provide health care to agricultural laborers around the country. These hospitals operate according to the government strategy for primary care development and are responsible for health services such as health promotion, medical treatment, disease control and prevention, rehabilitation and consumer protection [11]. However, some studies have shown that HPH services are limited owing to insufficient personnel. Screening tests such as urinalysis are important for health monitoring. However, most HPHs do not provide routine urinalysis examinations. Remarkably, most HPH patients work in the agricultural sector. These farmers are at increasing risk of exposure to occupational hazards. Therefore, long-term provision of health services for agricultural workers must be tailored to each community’s needs and health problems. This responsibility is in accord with the strategy to develop HPHs in Thailand and to promote the role of HPHs in coordinating with various local community agencies to address health problems and obtain full benefits for the community [11].

Nan Province is situated in the north of Thailand. The province—particularly Wiangsa District—is a main rice farming area in the country. In Nan Province, 9900 rai are used for rice farming (1 rai = 1600 square meters), which is more than 80% of the total provincial area [8,12]. Among the agricultural highlands in Thailand, the central area of Nan Province is highly productive for rice farming. The target group of the present study was female rice farmers in Baan Srinachuen, Thungsrithong Subdistrict, Wiangsa District, Nan Province. Female farmers are considered an important source of labor in the local area. Increased attention is currently being paid to the role of women in agriculture in the global labor market. Therefore, the introduction of active health services to local agricultural workers in this area is important. The study area of the present study was Moo 4 Baan Srinachuen, Thungsrithong Subdistrict, Wiangsa District, Nan Province. This area contains the highest number of rice farmers in the province and is served by a subdistrict HPH, which acts as a network health service for local agricultural workers. This study focused on assessing working conditions among female rice farmers and used urinalysis to determine renal function. The renal system is responsible for maintaining the balance of water and urinary pH, regulates blood pressure and produces important hormones. Impaired renal function is associated with the development of several diseases. There are many studies on the prevalence of health risks in farmers in Thailand. For example, Laohaudomchok et al. examined health risks associated with pesticide use in Thailand and Kongtip et al. investigated the occupational health of informal workers in Thailand. The findings of these studies are consistent with the 2017 Occupational and Environmental Disease Situation Report, which found that Nan Province had the second highest heat-related disease rate in northern Thailand (0.21 per 100,000 population) [13,14,15]. Therefore, female farmers should receive early renal screening. The study findings may provide a better understanding of female farmers’ working conditions and health and assist the subdistrict HPH to plan strategies to improve the health of female agricultural workers.

## 2. Materials and Methods

### 2.1. Study Design and Participants

In this cross-sectional mixed methods study, data collection was conducted during April–June 2021. The study population was 183 rice farmers in Baan Srinachuen, Thungsrithong Subdistrict, Wiangsa District, Nan Province [16]. The population size was used to estimate the required sample size [17]; this was increased by 5% to take account of dropout rates, producing a required sample size of 65. Purposive sampling was used to select female rice farmers. The inclusion criteria were as follows: (1) rice farmers aged over 20 years involved in all aspects of rice growing (i.e., preparing the ground, planting, plant management, harvesting); (2) no less than 1–3 years of rice planting experience; (3) ability to communicate in Thai; and (4) provision of voluntary consent to participate in the study.

### 2.2. Data Collection Instrument

The study aim was to identify hazards that the rice farmers were exposed to in their farming activities and to assess their health status using urinalysis.

Accordingly, the primary data were collected using in-depth interviews to obtain information about participants’ demographic characteristics. In addition, a walk-through survey was performed in the field to collect data on the hazards involved in the four stages of rice farming: preparing the ground, planting, plant management and harvesting.

In the urinalysis, a chemical examination was conducted using a urine dipstick (CYBOWTM, DFI Co., Ltd., Jillye-myeon, Korea). The analysis examined seven parameters: specific gravity (SG), pH, blood, glucose, protein, nitrite and leukocytes for primary screening. Disease diagnosis was also conducted on the rice farmers.

### 2.3. Data Acquisition and Analysis

A walk-through survey and interviews were used to collect data about risks and hazards in the different stages of farming activities. A 30–45 min interview was conducted in the local dialect with each participant. Farmers were asked about their sociodemographic characteristics, such as status, age, education and length of current occupation.

In the urinalysis, the collected urine samples were analyzed using chemical and visual examinations. The urine sampling and analysis were performed by a professional medical technologist. Samples were compared with the normal urine SG range of 1.010–1.030 and normal pH range of 5–9. Negative results for the blood, glucose, protein, nitrite and leukocyte tests were regarded as normal. The urine dipstick test followed the protocol for the analysis of the urine sample. The urine sampling method, read time and accuracy of the results were carefully considered.

### 2.4. Ethics Approval

Urine sampling is considered a non-invasive method with minimal risk. Therefore, subjects were not exposed to toxic chemicals during the dipstick urine test. This study was evaluated and approved by the Ethical Review Committee for Human Research of the Faculty of Public Health, Mahidol University (project research number MUPH 2021-023) and MUPH 2021-014).

The research team introduced themselves to the participants and explained the sample collection, research objectives, research procedure, participants’ rights regarding consent and participation and their right to choose whether to accept or refuse research participation. The researchers allowed participants to make their own decisions about participation. Individuals who agreed to participate in the study provided written informed consent. The data collected were kept confidential and anonymous and only used or presented for academic purposes.

### 2.5. Statistical Analysis

Descriptive statistics—frequency and percentage—were calculated to analyze the participants’ demographic characteristics. The urinalyses were performed according to the standards of the Faculty of Medicine, Siriraj Hospital, Mahidol University [18].

## 3. Results

### 3.1. Characteristics of the Participants

Demographic characteristics of the 65 female rice farmers are shown in Table 1. Most respondents were aged 50–59 years (46.2%) and most had only primary school education (78.5%). Most participants were married (90.8%). Most had been working in their current occupation for 31–40 years (27.7%). Regarding smoking and alcohol consumption, most participants did not smoke cigarettes (98.5%) but did drink alcohol (38.5%).

### 3.2. Risks and Hazards in the Rice Farming Operation

The results of the interview and the walk-through survey on the risks and hazards in rice farming procedures are shown in Table 2. The walk-through survey results identified both short-term safety risks, such as cuts and falls and long-term safety risks, such as chemical and ergonomic hazards. The risks and hazards are summarized below according to the four stages of rice farming: (1) preparing the ground, (2) planting, (3) plant management and (4) harvesting.

Stage 1: preparing the ground

This stage involves ground preparation by manually clearing the land with knifes, pesticides or occasional burning. The land is subsequently plowed, harrowed and irrigated to retain water in the land plots for some time before harrowing the plots with tractors. In the pre-planting stage, the main risk to farmers is exposure to physical hazards from the use of agricultural vehicles or machinery, as well as continuous heat contact from sunlight during their work on the farm.

Stage 2: planting

In this stage, water is irrigated into the land plots until the required water level is reached. When ready, the rice crops are manually transplanted in the appropriate season. The main risks in this stage are ergonomic hazards from the awkward postures adopted when lifting, moving and planting the crops and the risk of slipping on the rough land surface. Another inevitable risk is standing in waterlogged ground and coming into contact with various bacteria. This risk is higher for farmers with open wounds. Other risks are contact with poisonous insects or animals.

Stage 3: plant management

After the rice planting period, the farmers have to ensure the plantation quality by removing weeds, spraying chemicals and adding fertilizers. The main risk in weed elimination and pest prevention is contact with chemical hazards from mixing, spraying and storing chemical substances. There is also the problem of contact with chemical contaminants, which poses a risk to families and communities.

Stage 4: harvesting

In the harvesting stage, farmers use sickles to harvest the rice. The sickled rice is dried in the rice fields until it is completely dry, before being transported for threshing. This procedure uses either manual labor or threshing machines. After the threshing stage, the rice paddies are moved to the rice barns, which are cleaned in advance using mainly chemical sprays. In this stage, the main risk to farmers is from the physical hazard of contact with sharp agricultural machines, equipment or tools, as well as continuous heat exposure in the work environment. Moreover, farmers may come into contact with chemical hazards from dust and rice straw from the threshing wastes and chemicals used in cleaning the rice barns.

### 3.3. Health Status Examination Using Urinalysis (Dipstick Test)

Urinalysis was performed on 65 female rice farmers in Nan Province using chemical examination of seven parameters: SG, pH, blood, glucose, protein, nitrite and leukocytes. In descending order, negative test results include glucose in 65 cases (100.0%), nitrite in 65 cases (100.0%), blood in 52 cases (80.0%), protein in 51 cases (78.5%) and leukocytes in 50 cases (76.9%). Most participants (38.5%) had an SG of 1.030 and most (83.0%) had a pH of 5–6, as shown in Table 3.

## 4. Discussion

The target group of the present study was female rice farmers in Baan Srinachuen, Thungsrithong Subdistrict, Wiangsa District, Nan Province. Female farmers are considered an important source of labor in the local area. More attention is currently being paid to the role of women in agriculture in the global labor market. According to the ILO, women working in agriculture have a high incidence of injury and disease. Exposure to pesticides and mixing or applying other harmful agrochemicals are the main occupational risks. Other hazards are contact with biological agents, which cause allergies, infections and parasitic diseases. Other effects from exposure to hazards are noise-induced hearing loss, musculoskeletal disorders (such as repetitive stress), injuries and back pain. Women in developing countries are particularly prone to such risks because they have insufficient education to protect themselves from occupational health problems [19].

Health threats from the working environment and unsafe working conditions are present in all four stages of rice farming: (1) preparing the ground, (2) planting, (3) plant management and (4) harvesting. In all these stages, farmers may experience four risk factors: physical hazards, chemical hazards, biological hazards and ergonomic hazards. The results of the analysis of hazards in each stage can be summarized as follows.

In the ground preparation stage, the main risk is from the physical hazard of using agricultural vehicles and machines. Farmers who are unfamiliar with using modern or advanced machinery are more at risk of injuries [20]. Moreover, they are inevitably at risk of continuous heat exposure from sunlight during their work. Heat exposure in agricultural work can lead to heat stress, a situation in which the body overheats and is unable to cool itself. Heat stress may also be caused by high temperatures, high moisture, strong sunlight and heavy work burden [21,22]. We used secondary data from the Nan Province Meteorological Department to examine climatic conditions during the test period. During this period, the temperature and humidity in Nan Province were high (temperature 28.83 °C, humidity 75.83%, air velocity 15.47 km/h) [23]. Some studies report that occupational heat stress is associated with a higher rate of kidney disease [24].

The main risk in the planting stage is from ergonomic hazards owing to use of awkward postures when lifting and moving objects/rice when planting and the risk of slipping when working on unstable ground. This is consistent with findings that farming activities such as carrying and lifting bags of rice or fertilizer are repetitive and involve awkward postures, which lead to health problems in farmers [9]. Another inevitable risk is biological hazards from standing in waterlogged rice fields for a long time. This increases the chance of contact with bacteria, a risk that is higher for farmers with open wounds. Because farmers are in close contact with animals and plants, they are likely to encounter poisonous insects or animals. Farmers may have direct contact with environmental pathogens, fungi, infected animals and allergenic plants [25].

In the plant management stage, the main risk is from chemical hazards when mixing, spraying and storing chemicals. The use of chemical pesticides in rice farming is recent; however, their use has rapidly increased and has had substantial negative effects on human health and the environment [26]. This is consistent with a report that farmers in the Philippines experience acute and chronic negative health effects from using chemical pesticides [27].

In the harvesting stage, the main physical hazards farmers are exposed to are sharp agricultural machines, tools and objects, as well as continuous heat exposure. A previous study showed that Thai rice farmers are exposed to sunlight for long periods; such exposure causes health problems such as weakness and stress [10]. During harvesting, farmers are also exposed to chemical hazards from dust and rice straw from threshing wastes and chemicals used to clean the rice barns. Farmers may use hazardous chemical substances in the form of liquids, concentrates, powders, dust, particles, aerosols and fogs. These chemicals enter the environment through water, wind and the absorption process [28].

The urinalysis (using a dipstick test) of 65 female rice farmers in this study showed that chemical urinalysis is an important and useful way of diagnosing and treating disease and performing follow-up health checkups when necessary. The present test results showed that 38.50% of participants had high SG (1.030); this indicates more concentrated urine, which can be caused by dehydration from sweating or a clinically dehydrated state [29]. Another possible cause of high SG urine is the presence of additional substances in urine such as protein, blood and leukocytes (Table 3). Moreover, dehydration plays a role in kidney stone formation [24]. The Occupational Safety and Health Administration recommends that, when working in a heat index of 32.78–39.44 °C, workers should be reminded to drink more often (about four cups/hour). In addition, frequent breaks in a cool shaded area should be scheduled [30]. A urine pH of 5–6 was found in 83% of participants. This indicates that the urine was normal and slightly acidic. This is in accord with findings from studies of Thai sugarcane workers, who tended to have acidic urine (pH 5–6) [31]. We also tested whether age and work experience were associated with urine pH level and specific gravity. We found no statistically significant difference among the variables. It might probably be due to a small sample size. The urine of some female rice farmers in this study contained protein, blood and leukocytes. These results are in accord with a report that the urine of sugarcane cutters in Nicaragua usually contains protein, blood and leukocytes [32]. The present tests showed negative (i.e., normal) results for glucose and nitrite.

### Limitations

The present study has some limitations. The data collection period was at the beginning of the COVID-19 pandemic in Thailand. Because of time limitations, the sample size was limited and chemical exposures were not identified. However, the data reflect real working conditions. Future studies are needed that compare female farmers with female non-farmers and that have larger samples, more varied study areas, chemicals exposures and different groups of agricultural workers to compare the risks involved in different occupations.

## 5. Conclusions

The present study showed that female rice farmers are at risk of exposure to physical, chemical, biological and ergonomic hazards during the different stages of rice farming. The urinalysis using a dipstick test showed a tendency for high SG, but normal urinary pH. The test results were positive in some female rice farmers for protein, blood and leukocytes. These cases were subsequently followed-up for treatment. Farmers with leukocytes in the urine should be treated for inflammation of the urinary tract or kidney [33]. Blood in the urine can originate from many sites, including the kidney, ureter, bladder, urethra or other urogenital tract structures [34]. If health outcomes are not monitored, kidney function abnormalities can lead to untreated health conditions, permanent damage or loss of life. The findings of this preliminary study need to be confirmed, but they suggest that relevant local agencies, particularly primary health care services, should prioritize active and continuous health care and health surveillance for farmers. This would help to reduce occupational risk factors and promote good health and safety in farming activities.

## Figures and Tables

**Table 1:** Population characteristics of female rice farmers (*n* = 65).

Variables		Frequency (*n* = 65)	Percentage
Age	Mean (SD)	52.1 (9.1)	
	(Min–max) (year)	(31–68)	
	30–39	6	9.2
	40–49	18	27.7
	50–59	30	46.2
	≥60	11	16.9
Education Level	Primary	51	78.5
	Secondary	5	7.7
	High school	6	9.2
	Vocational/high vocational	2	3.1
	Bachelor’s degree	1	1.5
Marital status	Single	3	4.6
	Married	59	90.8
	Divorced or widowed	3	4.6
Work experience	Mean experience (SD)	28.14 (14.20)	
	(Min–max) (year)	(1–50)	
	≤10	11	16.9
	11–20	16	24.6
	21–30	11	16.9
	31–40	18	27.7
	≥41	9	13.9
Smoking	No	64	98.5
	Yes	1	1.5
Alcohol drinking	No	40	61.5
	Yes	25	38.5

SD, standard deviation.

**Table 2:** Risks and hazards in the rice farm operations.

Process	Hazards	Risk
1. Preparing the ground -Chemical use, burning to eliminate weeds and ground preparation -Land preparation (plowing and harrowing) 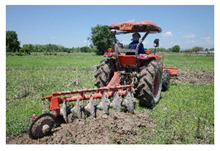 Field research, 2021 Arphorn et al., 2021	Physical	-Risks of cutting or stabbing by tools or objects -Exposure to sunlight and other extreme weather conditions -Noise of the agricultural machines
	Chemical	-Contact with dust and smoke from burning for land preparation -Contact with chemicals used for soil improvement
	Biological	-Contact with dangerous plants or animals
	Ergonomic	-Awkward posture
2. Planting -Water irrigation of the land plots and water control to required level -Rice crop planting or sowing 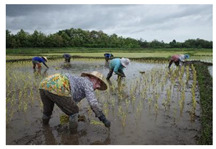 Field research, 2021 Arphorn et al., 2021	Physical	-Exposure to sunlight and other extreme weather conditions
	Chemical	-Contact with chemical substances used for soil improvement
	Biological	-Contact with bacteria owing to weather conditions and soil moisture -Contact with dangerous plants or animals
	Ergonomic	-Long working hours -Head and back fatigue from bending in awkward postures -Repetitive work -Slipping in the rice fields
3. Plant management -Use of fertilizers or hormones -Spraying pesticides and herbicides 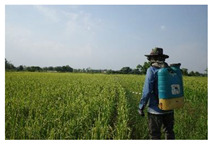 Field research, 2021 Arphorn et al., 2021	Physical	-Exposure to sunlight and other extreme weather conditions
	Chemical	-Contact with chemical substances from fertilizers, pesticides and herbicides
	Biological	-Contact with bacteria owing to weather conditions and soil moisture -Contact with dangerous plants or animals
	Ergonomic	-Awkward postures when lifting and moving objects and spraying of pesticides and herbicides -Repetitive movement when spraying chemicals -Slipping in the rice fields
4. Harvesting -Rice harvesting -Drying or sun drying to reduce the paddy moisture -Rice threshing or hitting -Removal of waste or rice straw contamination -Rice storage or packaging for transportation 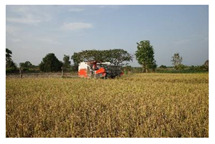 Field research, 2020 Arphorn et al., 2021	Physical	-Accidents from agricultural vehicles or machines
		-Cutting and stabbing risks from sharp objects or tools
		-Noise of agricultural machines
		-Exposure to sun and other extreme weather conditions
	Chemical	-Contact with dust from rice threshing
		-Contact with dust, rice straw or agricultural waste
		-Contact with chemical substances used to clean the rice barns
	Biological	-Contact with bacteria owing to weather conditions and soil moisture
		-Skin irritation from contact with rice paddies
	Ergonomic	-Awkward postures in moving objects, equipment and products
		-Excessive force in rice threshing or hitting

**Table 3:** Urinalysis dipstick test results for female rice farmers (*n* = 65).

Parameter		Frequency (*n* = 65)	Percentage
Specific Gravity	≤1.010	2	3.1
	1.015	4	6.1
	1.020	15	23.1
	1.025	19	29.2
	1.030	25	38.5
pH	5–6	54	83.0
	6.5–7	10	15.5
	8–9	1	1.5
Blood	Negative	52	80.0
	Positive	13	20.0
Glucose	Negative	65	100.0
	Positive	-	-
Protein	Negative	51	78.5
	Positive	14	21.5
Nitrite	Negative	65	100.0
	Positive	-	-
Leukocytes	Negative	50	76.9
	Positive	15	23.1

## Data Availability

The data presented in this study are available from the corresponding author on reasonable request. The data are not publicly available to protect the participants.
